# The use of Heated, Humidified, High-flow Nasal Cannulas, and Length of Hospital Stay Among Extremely Preterm Infants

**DOI:** 10.2174/0118743064336750241115115435

**Published:** 2024-11-25

**Authors:** Tareq F. Alotaibi, Dana Alzahrani, Fawzeah Alenazi, Sarah Altokhais, Afnan Slemani, Kamal Ali, Raghad Alzahrani, Abdullah M. Alanazi, Taha Ismaeil, Mohammed Alqahtani, Saif Alsaif, Saleh S. Algarni

**Affiliations:** 1 Department of Respiratory Therapy, College of Applied Medical Sciences, King Saud bin Abdul Aziz University for Health Sciences, Riyadh, Saudi Arabia; 2 King Abdullah International Medical Research Center, Riyadh, Saudi Arabia; 3 Neonatal Intensive Care Department, King Abdulaziz Medical City, Ministry of National Guard Health Affairs, Riyadh, Saudi Arabia; 4 Department of Respiratory Services, King Abdulaziz Medical City, Ministry of National Guard Health Affairs, Riyadh, Saudi Arabia; 5 Department of Cardiovascular Technology, College of Applied Medical Sciences, King Saud bin Abdulaziz University for Health Sciences, Riyadh, Saudi Arabia

**Keywords:** Extremely preterm infants, Heated, Humidified, High-flow nasal cannula, Length of hospital stay, Neonatal intensive care unit

## Abstract

**Background:**

Extremely preterm infants (EPIs) often require advanced respiratory support to survive, and one such intervention is the heated, humidified, high-flow nasal cannula (HHHFNC). While the use of this cannula in EPIs has been studied, the relationship between its use and the length of hospital stay is an important yet unexplored research area that we aim to investigate in this study.

**Methods:**

In a quantitative retrospective cohort study, data were extracted from an electronic database. The study included all EPIs younger than 28 weeks of gestational age admitted to the neonatal intensive care unit of a tertiary hospital from January 1, 2020, to December 31, 2022. The descriptive analysis was conducted to describe each infant’s demographic, maternal, and neonatal characteristics. A chi-squared test was also conducted, and a *p*-value of < 0.05 was considered statistically significant.

**Results:**

The findings suggest that infants who receive an HHHFNC have a longer hospital stay than those who do not. Specifically, infants who did not receive a cannula spent 42.5 days on average in the hospital, compared with 99 days among those who received it, with a significant *p*-value (p=0.0001).

**Conclusion:**

Infants receiving a cannula stay in hospital on average for twice as long as those who do not. However, to reduce the possibility of bias and produce more reliable results, we advise conducting clinical trials or prospective studies in future research.

## 1 INTRODUCTION

Extremely preterm infants (EPIs) are defined as neonates with a low gestational age of less than 28 weeks [1]. They often face respiratory distress due to underdeveloped lungs and a deficiency of surfactant, which coats the inside of the lungs and helps keep them inflated. Without sufficient surfactant, the air sacs in the lungs can collapse, making it difficult for the infant to breathe [2]. Additionally, the muscles used for breathing are not fully developed in EPIs, which can lead to rapid, shallow breathing or periods of apnea (temporary cessation of breathing) [3]. Gestational age directly correlates with infants’ morbidity and mortality [4]. For infants born before 28 weeks of gestation, death and morbidity rates are considerably higher [5]. Healthcare providers can use various types of respiratory support to assist EPIs in breathing, including the heated, humidified, high-flow nasal cannula (HHHFNC), which is used to deliver heated and humidified medical gases into an EPI’s lungs through prongs placed in their nostrils [6]. The primary purpose of using an HHHFNC for this population is to provide non-invasive respiratory support, reducing the need for invasive mechanical ventilation (MV) and its potential complications, which include infection, bronchopulmonary dysplasia (BPD), and air leak syndromes [7].

The HHHFNC’s mechanism of action is the delivery of low levels of positive-end expiratory pressure to stabilize the upper airway and facilitate spontaneous breathing efforts [8]. Moreover, it provides an increased gas flow rate, promoting dead space washout and improving carbon dioxide clearance [6]. These features help to maintain adequate oxygenation and ventilation while preserving the infant’s natural mucociliary clearance mechanisms [9].

While the HHHFNC might be a safe and effective replacement for nasal continuous positive airway pressure (CPAP) for some newborns, there is a lack of information about how effective it is for EPIs [10]. Several studies investigated the efficacy of HHHFNCs in preterm infants [7, 9, 11-13]. For instance, a retrospective chart review [11] showed that using an HHHFNC is linked with a considerable increase in the time spent on respiratory support, a reduction in oral feeding at discharge, and lower rates of being discharged home. In another study [12] exploring the effect of the early use of a high-flow nasal cannula on the length of hospital stay, the group receiving the high-flow oxygen had significantly longer hospital stays than those who received standard oxygen. Another study [13] revealed similar results, finding a strong association between using an HHHFNC and longer hospital stays among pediatric asthma patients. On the other hand, a systematic review and meta-analysis [7] indicated that an HHHFNC significantly reduces the incidence of treatment failure and intubation rates compared to standard nasal cannulas or face masks among preterm infants requiring respiratory support. Another randomized controlled trial [9] derived similar findings, showing lower intubation rates among preterm infants treated with an HHHFNC than those receiving nasal CPAP. Therefore, this study aims to ascertain the correlation between using an HHHFNC and the length of hospital stay among EPIs.

## 2 MATERIALS AND METHODS

A quantitative retrospective cohort study was conducted in King Abdulaziz Medical City (KAMC), Riyadh, Kingdom of Saudi Arabia, from January, 2020, to December 31, 2022. Our sample comprised all newborn infants with a gestational age of < 28 weeks born at KAMC. The exclusion criteria included infants born with a gestational age of less than 23 weeks and those diagnosed with major congenital disorders. The final sample comprised 93 newborn infants.

### 2.1 Data Collection Methods, Instrument Used, and Measurements

The data were collected from the electronic medical records available on BESTCare, from which mothers’ and infants’ demographic data were extracted. Differences in mothers’ and infants’ prenatal and postpartum corti- costeroid use, birth weight, sex, mode of delivery, and gestational age at birth were examined. Apgar scores at 1 and 5 minutes after birth, as well as surfactant use in mechanically ventilated patients, were also recorded. Additionally, the length of hospital stay of the infants was recorded. Data on each infant’s use and duration of all respiratory support modes were collected, including CPAP, MV, HHHFNC therapy, and low-flow nasal cannula oxygen therapy. The level of respiratory support was graded from highest to lowest, whereby MV ventilation was considered the highest level of support, CPAP or an HHHFNC was considered moderate support, and low-flow nasal cannula ventilation was considered the lowest level of respiratory support. If the types of respiratory support varied on the same day, the highest-level advanced respiratory mode was recorded. If CPAP and an HHHFNC were used on the same day, detailed hours were calculated for each day, and modified hours per day were used for each method.

### 2.2 Data Management and Analysis Plan

All data management and statistical analyses were performed using Stata BE software (version 17: StataCorp LLC, TX, USA). Statistical significance was set at *p* < 0.05. Continuous variables were reported by distribution as the means (standard deviations) of normally distributed data or the medians (interquartile ranges; IQRs) of non-normally distributed data. Categorical data were presented as frequencies (percentages). All infants’ characteristics and outcomes according to their HHHFNC status were compared using chi-squared tests for categorical variables and the Mann-Whitney U-test for non-normally distributed continuous variables.

## 3 RESULTS

The study included 93 infants who were born at KAMC over the two years between January 1, 2020, and December 31, 2022. The demographics of the patients involved in this research are summarized in Table **1**. Overall, sixteen patients (17.2%) did not receive an HHHFNC, including four female patients (25%) and twelve male patients (75%). By contrast, 77 patients (82.8%) received an HHHFNC for at least one day, including 42 female patients (54.55) and 35 male patients (45.45%), with a *p*-value of 0.031. Overall, the average age of infants who received an HHHFNC for at least one day was 26 gestational weeks (25-27), with a *p*-value of 0.0080. The average birth weight of patients who did not receive an HHHFNC for at least one day was 657.5 g (range: 615-780 g), compared with 825 g (range: 700-960 g) among patients who received an HHHFNC for at least one day, with a *p*-value of 0.0285. The average maternal age of patients who did not receive an HHHFNC (31 years, range: 26-35 years) was similar to that of patients who received an HHHFNC for at least one day (31 years, range: 26-35 years), with a *p*-value of 0.9812. Furthermore, 39 infants (65%) who received an HHHFNC also received postnatal steroids, while six of the infants (37.50%) who did not receive an HHHFNC for at least one day received postnatal steroids, with a *p*-value of 0.219. Additionally, 69 infants (89.61%) who received an HHHFNC also received caffeine therapy, while fourteen of the infants (87.50%) who did not receive an HHHFNC received caffeine therapy, with a *p*-value of 0.545. Thirteen (81.25%) of the infants who received an HHHFNC also received surfactant therapy, as well as 70 (90.91%) among the infants who did not receive an HHHFNC, with a *p*-value of 0.187. Table **2** shows the number of BPD diagnoses among those who received an HHHFNC for at least one day. There were 50 infants (65%) who were diagnosed with BPD and received an HHHFNC, while four infants (25%) who were diagnosed with BPD did not receive an HHHFNC, with a *p*-value of 0.486. The relationship between length of hospital stay and HHHFNC use is shown in Table **3**, revealing that infants who did not receive an HHHFNC stayed in the hospital on average for 42.5 days (range: 4-97.5 days), while those who received an HHHFNC remained in the hospital on average for 99 days (range: 86-125 days), with a *p*-value of 0.0001.

These findings suggest that infants receiving an HHHFNC were thus statistically significantly associated with a longer hospitalization period.

## 4 DISCUSSION

The current study aims to find the correlation between the use of an HHHFNC and the length of hospital stay among EPIs in a single center over two years. The findings suggest that receiving an HHHFNC is statistically significantly associated with longer hospitalization.

Compared with our findings with recent research analyzing factors affecting the duration of hospital stay among very low birth weight infants [23] it was found that gestational age and type of management – such as CPAP – significantly prolonged the hospital stay. Similarly, our results demonstrate that the use of HHHFNCs – which is comparable to CPAP – extends the patient’s hospital stay. Thus, both management types could increase the hospital stay considering their substitute use in neonatal settings [23].

In our study, we found that the infants who received an HHHFNC had longer hospital stays than those who did not receive an HHHFNC. Based on previous studies, this might have been because preterm infants often have immature respiratory systems and are at a higher risk of developing respiratory distress syndrome, apnea of prematurity, and other respiratory complications [14]. These underlying respiratory issues increase the likelihood that preterm infants will require respiratory support such as an HHHFNC, which can lead to a longer hospital stay [14]. Furthermore, preterm infants who require an HHHFNC are generally sicker and have more severe respiratory problems compared to preterm infants who do not need this level of support [11]. Accordingly, the severity of the respiratory illness is a major factor contributing to longer hospital stays, as infants with more severe respiratory illness require more intensive monitoring and care [11]. Moreover, preterm infants often face additional challenges – such as difficulties with feeding, growth, and neurodevelopment progress [14] – which might also contribute to a longer hospital stay, as the medical team needs to monitor and manage these issues while providing the infant with respiratory support [14].

While the survival rates of EPIs have increased, it has increased the risk of experiencing complications related to their birth, meaning that establishing good ventilation is crucial for their survival [15]. Specifically, EPIs require extra care, given that an early gestational age affects spontaneous breathing and leads to respiratory distress [15]. According to the demographic and clinical characteristics of the patients involved in this research, we found that specific demographics influence clinical outcomes. Specifically, more female infants received an HHHFNC than male infants, suggesting that preterm female infants are sicker and need more respiratory support than their male counterparts. This differs from the results of a previous study [16], which mentioned that preterm female infants have better outcomes than male infants. We also noticed that infants with lower gestational ages and birth weights were less likely to receive an HHHFNC compared to heavier infants with higher gestational ages. Nonetheless, most infants diagnosed with BPD received an HHHFNC.

Our study yields noteworthy results with meaningful implications for future research and clinical practice. Our results suggest that while using an HHHFNC does not shorten the length of hospital stay, it appears to be safe and well tolerated among EPIs, with no increases in adverse events such as BPD or mortality [17]. This indicates that HHHFNCs are a feasible and safe non-invasive respiratory support option that is worth investigating in further studies [18]. We thus recommend that future research examines other factors that might influence the effect of HHHFNCs on the length of hospital stay, including comorbidities and concurrent therapies. In addition, evaluating the cost-effectiveness of using an HHHFNC *versus* conventional methods could provide essential insights for policymakers and healthcare administrators when considering resource allocation and guidelines for respiratory support in neonates [19]. Furthermore, prospective multicenter randomized controlled trials comparing different modes and flow rates of HHHFNCs are warranted to establish optimal parameters for various clinical scenarios and patient characteristics [20]. Regarding clinical practice, based on our results, neonatologists and nursing staff involved in caring for EPIs should consider implementing HHHFNCs as a first-line option for respiratory support whenever feasible [21]. This change in practice might result in a reduced overall morbidity rate associated with prolonged hospital stays while maintaining safety standards. Moreover, minimizing exposure to invasive procedures such as intubation and MV *via* the early use of an HHHFNC might promote faster recovery and improved neurodevelopmental outcomes [22]. However, the proper training and ongoing supervision of personnel skilled in operating HHHFNC devices remain vital components in facilitating their successful implementation within neonatal intensive care units. As new data emerge, regular updates to institutional policies and educational materials must reflect best practices for HHHFNC utilization [22]. To summarize, future investigations should continue exploring ways to enhance the application of HHHFNCs, and large-scale multicenter randomized controlled trials with consistent methodologies are necessary to evaluate the full impacts of HHHFNCs on the clinical outcomes of preterm infants [19]. Additionally, the prompt dissemination of knowledge gained from recent studies should encourage informed decision-making among those practitioners responsible for providing critical care services to vulnerable newborn populations.

## 5 LIMITATIONS

While this study obtained insightful outcomes by focusing on EPIs as an under-researched population, the retrospective cross-sectional design used might have resulted in bias and limitations in the accuracy of the data retrieved from the database system. Moreover, this study had a limited scope since we only had access to the data available at that time, therefore, we could not account for changes over time.

## CONCLUSION

This study has explored the association between the use of HHHFNCs and the length of hospital stay among EPIs, finding that infants who receive an HHHFNC stay in the hospital for twice as long as those who do not receive one. We recommend further investigations and clinical trials or prospective studies on this topic to eliminate the risk of bias and obtain more accurate results.

## Figures and Tables

**Table 1:** Demographic comparison between the use and non-use of heated, humidified, high-flow nasal cannulas (*n* = 93*).

**Demographic**		**Receipt of HHHFNC for At Least One Day**		***p*-value**
Gender	-	No (*n* = 16)	Yes (*n* = 77)	0.031
	F	4 (25%)	42 (54.55%)	
	M	12 (75%)	35 (45.45%)	
Gestational age in weeks	-	24 (23-26)	26 (25-27)	0.0080
Birth weight in grams	-	657.5 (615-780)	825 (700-960)	0.0285
Maternal age in years	-	31 (26-35)	31 (25-36)	0.9812
APGAR at 1 min	-	4 (3-6)	6 (4-7)	0.1325
APGAR at 5 min	-	7 (6-8)	8 (7-8)	0.1561
First ABG PH in the first 24 hours of life	-	7.21 (7.195-7.29)	7.26 (7.2-7.3)	0.0674
Presence of pregnancy complications	-	5 (31.25%)	42 (54.54%)	0.077
Delivery type	C-section	8 (50%)	41 (53.24%)	0.514
	Vaginal	8 (50%)	36 (46.75%)	
Receipt of at least one dose of ANS	-	11 (68.75%)	57 (74.02%)	0.439
Receipt of postnatal steroids	-	6 (37.50%)	39 (50.65%)	0.219
Receipt of caffeine therapy	-	14 (87.50%)	69 (89.61%)	0.545
Receipt of surfactant therapy	-	13 (81.25%)	70 (90.91%)	0.187

Note: * Total number of EPIs included in the study.

**Abbreviations: **HHHFNC: Heated, humidified, high-flow nasal cannula; F: Female, M: Male; APGAR: Appearance, pulse, grimace, activity, and respiration; ABG: Arterial blood gas; ANS: Antenatal steroids.

**Table 2:** Diagnosis of bronchopulmonary dysplasia and the use of heated, humidified high-flow nasal cannulas.

	Receipt of HHHFNC		*p*-value
Diagnosis of BPD	No (*n* = 16)	Yes *(n =* 77)	0.486
	4 (25%)	50 (65%)	

**Abbreviations: **BPD: Bronchopulmonary dysplasia; HHHFNC: Heated, humidified, high-flow nasal cannula.

**Table 3:** Length of hospital stay and the use of heated, humidified high-flow nasal cannulas.

	**Receipt of HHHFNC**		***p-*value**
Length of Hospital Stay in Days, Median (IQR)	No *(n =* 16)	Yes *(n =* 77)	0.0001
	42.5 (4-97.5)	99 (86-125)	

**Abbreviation: **HHHFNC: Heated, humidified, high-flow nasal cannula.

## Data Availability

The data supporting the findings of this article is owned by King Abdullah International Medical Research Center (KAIMRC) and is not publicly available. However, the data can be accessed upon reasonable request and with the necessary permissions from KAIMRC.

## LIST OF ABBREVIATIONS

EPIs: = Extremely Preterm Infants
HHHFNC: = Heated, Humidified, High-flow Nasal Cannula

## STANDARDS OF REPORTING

STROBE guidelines were followed.

## References

[1] (2023). World Health Organization (WHO). Preterm birth.. https://www.who.int/news-room/fact-sheets/detail/preterm-birth.

[2] Yadav S., Lee B., Kamity R. (2023). Neonatal respiratory distress syndrome. StatPearls..

[3] Triebwasser J.E., Treadwell M.C. (2017). Prenatal prediction of pulmonary hypoplasia.. Semin. Fetal Neonatal Med..

[4] Barros F.C., Rossello J.L.D., Matijasevich A., Dumith S.C., Barros A.J.D., dos Santos I.S., Mota D., Victora C.G. (2012). Gestational age at birth and morbidity, mortality, and growth in the first 4 years of life: findings from three birth cohorts in Southern Brazil.. BMC Pediatr..

[5] Ji X., Wu C., Chen M., Wu L., Li T., Miao Z., Lv Y., Ding H. (2022). Analysis of risk factors related to extremely and very preterm birth: a retrospective study.. BMC Pregnancy Childbirth.

[6] Jeon G.W. (2016). Respiratory support with heated humidified high flow nasal cannula in preterm infants.. Korean J. Pediatr..

[7] Jozwiak M., Millasseau S., Richard C., Monnet X., Mercado P., Dépret F., Alphonsine J.E., Teboul J.L., Chemla D. (2019). Validation and critical evaluation of the effective arterial elastance in critically ill patients.. Crit. Care Med..

[8] Guglielmo R.D., Hotz J.C., Ross P.A., Deakers T.W., Diep J.E.L., Newth C.J.L., Khemani R.G. (2022). High-flow nasal cannula reduces effort of breathing but not consistently *via* positive end-expiratory pressure.. Chest.

[9] Yoder B.A., Stoddard R.A., Li M., King J., Dirnberger D.R., Abbasi S. (2013). Heated, humidified high-flow nasal cannula *versus* nasal CPAP for respiratory support in neonates.. Pediatrics.

[10] Fleeman N., Dundar Y., Shah P.S., Shaw B.N.J. (2019). Heated humidified high-flow nasal cannula for preterm infants: an updated systematic review and meta-analysis.. Int. J. Technol. Assess. Health Care.

[11] Hoffman S.B., Terrell N., Driscoll C.H., Davis N.L. (2016). Impact of high-flow nasal cannula use on neonatal respiratory support patterns and length of stay.. Respir. Care.

[12] Franklin D., Babl F.E., George S., Oakley E., Borland M.L., Neutze J., Acworth J., Craig S., Jones M., Gannon B., Shellshear D., McCay H., Wallace A., Hoeppner T., Wildman M., Mattes J., Pham T.M.T., Miller L., Williams A., O’Brien S., Lawrence S., Bonisch M., Gibbons K., Moloney S., Waugh J., Hobbins S., Grew S., Fahy R., Dalziel S.R., Schibler A. (2023). Effect of early high-flow nasal oxygen *vs*. standard oxygen therapy on length of hospital stay in hospitalized children with acute hypoxemic respiratory failure: The PARIS-2 randomized clinical trial.. JAMA.

[13] Rogerson C., Owora A., He T., Carroll A., Schleyer T., AbuSultaneh S., Tu W., Mendonca E. (2023). High flow nasal cannula use is associated with increased hospital length of stay for pediatric asthma.. Pediatr. Pulmonol..

[14] Wang K., Zhao W., Li J., Shu W., Duan J. (2020). The experience of high-flow nasal cannula in hospitalized patients with 2019 novel coronavirus-infected pneumonia in two hospitals of Chongqing, China.. Ann. Intensive Care.

[15] Jobe A.H. (2012). What is RDS in 2012?. Early Hum. Dev..

[16] Cormack B.E., Harding J.E., Miller S.P., Bloomfield F.H. (2019). The influence of early nutrition on brain growth and neurodevelopment in extremely preterm babies: A narrative review.. Nutrients.

[17] Manley B.J., Owen L.S. (2016). High-flow nasal cannula: Mechanisms, evidence and recommendations.. Semin. Fetal Neonatal Med..

[18] Roca O., de Acilu M.G., Caralt B., Sacanell J., Masclans J.R., ICU collaborators (2015). Humidified high flow nasal cannula supportive therapy improves outcomes in lung transplant recipients readmitted to the intensive care unit because of acute respiratory failure.. Transplantation.

[19] Huang L., Roberts C.T., Manley B.J., Owen L.S., Davis P.G., Dalziel K.M. (2018). Cost-effectiveness analysis of nasal continuous positive airway pressure *versus* nasal high flow therapy as primary support for infants born preterm.. J. Pediatr..

[20] Kang W.Q., Xu B.L., Liu D.P., Zhang Y.D., Guo J., Li Z.H., Zhou Y.J., Xiong H. (2016). Efficacy of heated humidified high-flow nasal cannula in preterm infants aged less than 32 weeks after ventilator weaning.. Zhongguo Dang Dai Er Ke Za Zhi.

[21] Venanzi A., Di Filippo P., Santagata C., Di Pillo S., Chiarelli F., Attanasi M. (2022). Heated humidified high-flow nasal cannula in children: State of the art.. Biomedicines.

[22] Hochwald O., Osiovich H. (2010). The use of high flow nasal cannulae in neonatal intensive care units: Is clinical practice consistent with the evidence?. J. Neonatal Perinatal Med..

[23] Mehretie Y., Amare A.T., Getnet G.B., Mekonnen B. (2024). Length of hospital stay and factors associated with very-low-birth-weight preterm neonates surviving to discharge a cross-sectional study, 2022.. BMC Pediatr..

